# The Fingerprint of Anti-Bromodeoxyuridine Antibodies and Its Use for the Assessment of Their Affinity to 5-Bromo-2'-Deoxyuridine in Cellular DNA under Various Conditions

**DOI:** 10.1371/journal.pone.0132393

**Published:** 2015-07-10

**Authors:** Anna Ligasová, Radek Liboska, Ivan Rosenberg, Karel Koberna

**Affiliations:** 1 Institute of Molecular and Translational Medicine, Faculty of Medicine, Palacký University, Olomouc, Czech Republic; 2 Oligonucleotide group, Institute of Organic Chemistry and Biochemistry CAS, v.v.i., Prague, Czech Republic; Bascom Palmer Eye Institute, University of Miami School of Medicine;, UNITED STATES

## Abstract

We have developed a simple system for the analysis of the affinity of anti-bromodeoxyuridine antibodies. The system is based on the anchored oligonucleotides containing 5-bromo-2'-deoxyuridine (BrdU) at three different positions. It allows a reliable estimation of the reactivity of particular clones of monoclonal anti-bromodeoxyuridine antibodies with BrdU in fixed and permeabilized cells. Using oligonucleotide probes and four different protocols for the detection of BrdU incorporated in cellular DNA, we identified two antibody clones that evinced sufficient reactivity to BrdU in all the tested protocols. One of these clones exhibited higher reactivity to 5-iodo-2'-deoxyuridine (IdU) than to BrdU. It allowed us to increase the sensitivity of the used protocols without a negative effect on the cell physiology as the cytotoxicity of IdU was comparable with BrdU and negligible when compared to 5-ethynyl-2'-deoxyuridine. The combination of IdU and the improved protocol for oxidative degradation of DNA provided a sensitive and reliable approach for the situations when the low degradation of DNA and high BrdU signal is a priority.

## Introduction

5-Bromo-2'-deoxyuridine (BrdU) is commonly used for the detection of the cells in the S phase of the cell cycle [1–4]. This analogue of 2'-deoxyuridine is effectively incorporated in newly synthesised DNA by cellular DNA polymerases. Its detection is performed by means of special, anti-bromodeoxyuridine, antibodies. BrdU detection commonly requires additional steps to reveal the BrdU in DNA as it is hidden in the chromatin structure and is not accessible for an antibody reaction. Such treatments, however, usually result in the damage of many cellular components [1–7].

Probably the most widely used alternative approach is based on the use of 5-ethynyl-2'-deoxyuridine (EdU; [8]). The incorporated EdU is subsequently detected using the click reaction—a reaction catalysed by monovalent copper ions [8]. The approach based on EdU incorporation is quick and simple as no additional steps are needed. On the other hand, under common click reaction conditions reactive oxygen species are generated [9] which can negatively influence the detection e.g. GFP-like proteins and therefore the addition of oxygen-scavenger systems is required in such cases [10]. In addition, after prolonged pulses of EdU its toxicity has to be taken into account. Already submicromolar concentrations can lead to changes in the cell cycle progression as EdU induces damage of DNA and effectively inhibits thymidylate synthase leading to an imbalance of nucleoside and nucleotide pools [11–17]. These effects can finally result in cell death.

Another approach is based on the use of labelled nucleotides in the form of triphosphates and their introduction in cells e.g. by microinjection techniques (e.g. [18]) or by hypotonic treatment [19–21]. Although these systems usually do not disturb the cell structure, they do not allow the accurate control of the labelling time. Moreover, the microinjection techniques are relatively time-consuming, require special equipment and cannot be used if a very high number of labelled cells is necessary. In this respect, the techniques based on BrdU are still an important tool for cell cycle analysis and studies focused on DNA replication and chromatin organization.

There are a high number of monoclonal antibody clones available for BrdU detection on the market. Most of them are produced by mouse cells. Although it is obvious that particular antibody clones differ in their ability to detect BrdU incorporated in cellular DNA under various conditions, such comparison experiments are time consuming. It arises from the high number of BrdU detection systems. Probably the most frequently used system is based on acid treatment [2–5]. The concentrations of acid enabling the effective detection of BrdU in DNA structure by anti-bromodeoxyuridine antibodies vary between 1 and 4 M [2–5]. Moreover, according to our observations the obtained BrdU signal depends also on the incubation time and temperature.

Other protocols are based on the partial degradation of DNA by enzymatic approaches, alkali treatment or oxidative degradation of DNA in the presence of copper(I) ions [2,5,22]. It is evident that the consideration of which antibody is the best choice in the specific situation is relatively difficult. Although some information is available in the literature, it usually reflects experience with an individual clone in a specific situation rather than a detailed analysis of various clones under different conditions.

In the study presented here, we have developed a system enabling the fast comparison of the affinity of various antibody clones raised against BrdU. The system is based on the use of biotinylated oligonucleotides containing BrdU at three different positions. The oligonucleotides were anchored to the streptavidin coated surface and the affinity of six different monoclonal anti-bromodeoxyuridine antibody clones was tested. The EC50 and affinity constants were calculated for every oligonucleotide. The tests showed that every clone exhibited a different pattern of its affinity constant to the tested oligonucleotides (its fingerprint). The simultaneously performed analysis of the BrdU-derived signal in replicated cells using these antibodies and four different protocols of BrdU detection showed that the analysis of the fingerprints can serve as a reliable guide for the estimation of the reactivity of the clone with the incorporated BrdU in fixed and permeabilized cells. Interestingly, only two tested clones were suitable for the detection of BrdU in all the tested protocols. One of these two clones showed a higher reactivity with 5-iodo-2'-deoxyuridine (IdU) than to BrdU. In this respect, we showed that IdU exhibited a similar cytotoxicity as BrdU and much lower than EdU. We also improved the protocol based on the cleavage of DNA by means of oxidative degradation. The improvement of the composition of the cleavage solution resulted in the stabilization of the copper(I) solution without any effect on the BrdU signal intensity. When compared to the rest of the protocols used, it does not cause a considerable loss of DNA and in combination with IdU it provides sensitivity sufficient for the analysis of the cell cycle.

## Materials and Methods

### Cell cultures

Human HeLa cells (cervix, adenocarcinoma; a generous gift from Dr. David Staněk, Institute of Molecular Genetics, Prague; [11]) and HCT116 cells (colon, colorectal carcinoma; a generous gift from Doc. Marián Hajdúch, Palacký University, Olomouc; [11]) were used. The HeLa cells were cultivated in Dulbecco’s modified Eagle’s medium (DMEM, Gibco) supplemented with 10% foetal bovine serum (PAA Laboratories), 3.7 g/l of sodium bicarbonate and 50 μg/ml of gentamicin. HCT116 cells were grown in McCoy’s medium (Sigma Aldrich) supplemented with 10% foetal bovine serum (PAA Laboratories), 2.2 g/l of sodium bicarbonate and 50 μg/ml of gentamicin. The cells were cultured on coverslips (12 mm in diameter) in a Petri dish or in 96 flat bottom well plates (Orange Scientific) at 37°C in a humidified atmosphere containing 5% CO_2_.

### Oligonucleotide probes

The oligonucleotide probes were purchased from Generi-Biotech. In our experiments, we used biotinylated oligonucleotides with the following sequences:

5'-Biotin-GTTGCCTTAGGTTTTTCGTCGA-**BrdU**-3'–oligonucleotide with BrdU at the 3' end

5'-**BrdU**-TTGCCTTAGGTTTTTCGTCGAC-Biotin-3'—oligonucleotide with BrdU at the 5' end

5'-Biotin-GTTGCCTTAGGTTT-**BrdU-**TCGTCGAC-3'—oligonucleotide with central BrdU

5'-Biotin-GTTGCCTTAGGTTTTTCGTCGAC-3'–control oligonucleotide without BrdU

### Antibody used

The following monoclonal anti-bromodeoxyuridine antibody clones were tested in this study: BMC9318 (Roche), Bu-33 (Sigma Aldrich), B44 (Becton Dickinson), Bu6-4 (Genetex), Bu20a (BioLegend) and Bu5.1 (Millipore). As a secondary antibody, we used anti-mouse antibody conjugated with the fluorochrome Alexa Fluor 488 (1:100, Jackson ImmunoResearch).

### Optimal procedure for BrdU detection in oligonucleotides

All the incubation steps were performed on the laboratory shaker (300 rpm). We used black high binding 384-well plates in our experiments (Greiner Bio-One). The plates were incubated with 1 μg/ml streptavidin diluted in 1x PBS (20 μl per well) on the laboratory shaker at 4°C for 12 hours. Then, the temperature was increased to 37°C and incubation proceeded for another 30 minutes. Further, we added 100 μl of 1% BSA (bovine serum albumin) per well and incubated the plates for 1 hr. at 37°C. The plates were washed four times with a washing buffer consisting of 25 mM Tris-HCl, pH ~7.2 at 25°C, 150 mM NaCl, 0.1% BSA, and 0.05% Tween (TBT). After washing, the plates were incubated with 20 μl of 0.5 μM oligonucleotides at 24°C for 2 hrs. Next, the plates were washed with TBT and the primary anti-bromodeoxyuridine antibodies were applied for 2 hrs at 24°C. Serial two-fold dilutions of the antibodies were used starting from 0.039 μg/ml to 80 μg/ml. After washing, the well plates were incubated with Alexa Fluor 488 anti-mouse antibody (1:100 in TBT, 2 hrs, 24°C). The secondary antibody was washed out and the signal was measured using a PerkinElmer EnVision Plate Reader (Perkin Elmer). The data were analysed using Microsoft Excel and Sigma Plot software. The measurements were performed for the three independent experiments. The data are presented as mean values ± SEM.

### BrdU/IdU/CldU detection

The cells were incubated on glass coverslips in a Petri dish at 37°C in a humidified atmosphere containing 5% CO_2_ with or without 10 μM BrdU, IdU or 5-chloro-2'-deoxyuridine (CldU) for 20 minutes or 1 hour. After 10-minute fixation with 2% formaldehyde and permeabilization in 0.1% Triton X-100 in PBS, the incorporated BrdU/IdU/CldU was revealed using the below-mentioned protocols.

### Hydrochloric acid treatment

The cells were incubated either with 2N HCl or 4N HCl for 20 minutes at 24°C. The cells were then washed with 1x PBS and TBT and the incorporated BrdU was visualised using indirect immunofluorescence using primary and secondary antibodies (30 minutes each). The DNA was stained with DAPI (10 μM, 30 min, 24°C).

### DNase I treatment

The cells were washed with 10 mM Tris-HCl, pH 7.5 at 25°C and then incubated with the solution of primary antibody supplemented with 20 U/ml DNase I in 1x buffer for DNase I (10 mM Tris-HCl; pH 7.5 at 25°C, 2.5 mM MgCl_2_, 0.1 mM CaCl_2_). for 30 minutes at 37°C [5,23]. After washing with a TBT, the cells were incubated with a secondary antibody and DAPI for 30 minutes.

### Copper(I) treatment

The cells grown on coverslips in Petri dishes (diameter 60 mm) were washed three times with 1x PBS and then once with 2 ml of solution of 20 mM sodium ascorbate and 40 mM glycine (SAG solution, stored at -20°C). Then, 2 ml of SAG solution was exchanged for new 2 ml of SAG solution and immediately 2 ml of solution of 8 mM CuSO_4_ and 200 mM NaCl (stored at 4°C) was added. The samples were incubated on the shaker at 25°C and 300 rpm for 10 minutes. The cleavage solution should gradually turn light blue without the formation of any precipitate. The cells were then washed three times with 100 mM Tris-HCl, pH ~7.5 at 25°C. The last washing step was performed at 25°C and 300 rpm for 10 minutes. The cells were further incubated with the primary antibody supplemented with exonuclease III (1U/μl, Fermentas) in 1x buffer for exonuclease III (66 mM Tris-HCl, pH 8.0, 0.66 mM MgCl_2_) at 24°C for 30 min followed by washing with 25 mM Tris-HCl, ~pH 7.5 and 150 mM NaCl,. Next, samples were incubated with a secondary antibody with DAPI (30 min) in the same buffer.

### MTT assay

The MTT assay was performed according to the reported studies [11,24]. Briefly, the cells were seeded at the density of 5 × 10^3^ cells per well in 96 well plates and incubated at 37°C in a humidified atmosphere containing 5% CO_2_ for 24 hours. The tested nucleosides were added to the culture media and the cells were further incubated for 48 hours. Serial five-fold dilutions of IdU, CldU and BrdU were used starting at 0.00064 μM and ending at 50 μM. Then, the culture media were exchanged for nucleoside-free media and the cells were incubated for an additional 72 hours. The freshly-prepared 1 mM 3-(4,5-dimethylthiazol-2-yl)-2,5-diphenyltetrazolium bromide (MTT, Life Sciences) was added and the samples were incubated for 3 hours at 37°C in a humidified atmosphere containing 5% CO_2_. The culture media were removed (except 25 μl) and 50 μl of DMSO was added to each well. The samples were incubated for 10 minutes at 37°C and 300 rpm in the Thermomixer chamber (Eppendorf). Absorbance was measured using a PerkinElmer EnVision Plate Reader (Perkin Elmer) at 540 nm. The measurements were performed for three independent experiments and the data were evaluated using Sigma Plot. The data are presented as mean values ± SEM.

## Results

### The description of the system for antibody analysis

We have used synthetic biotinylated oligonucleotides modified with BrdU for an affinity analysis of the particular anti-bromodeoxyuridine antibody clones. BrdU was alternatively placed at 5' or 3' end or in the central part of the oligonucleotide chain. In the control experiments, we used biotinylated oligonucleotide without BrdU. The biotinylated oligonucleotides were anchored to the streptavidin-coated well plates. After that, we performed a reaction with the serial two-fold diluted mouse clones of anti-bromodeoxyuridine antibodies followed by the detection with a secondary anti-mouse antibody conjugated with Alexa Fluor 488. We tested several parameters during the optimisation of the procedure: the incubation time, concentration of streptavidin, concentration of oligonucleotide probes and concentration of the secondary antibody.

The optimal protocol with respect to the used buffers, well plates and temperatures was comprised of the following steps (The more detailed procedure is described in the Materials and Methods section): (1) binding of streptavidin to the surface of high binding 384-well plates at 4°C for 12 hours followed by a 30-min. incubation at 37°C; (2) incubation with 1% BSA for 1 hour at 37°C; (3) washing with TBT; (4) incubation with oligonucleotide probes for 2 hrs at 24°C; (5) incubation with anti-bromodeoxyuridine antibodies for 2 hrs at 24°C; (6) washing with TBT; (7) incubation with the secondary antibody for 2 hrs at 24°C and (8) washing with TBT.

**Fig 1:**
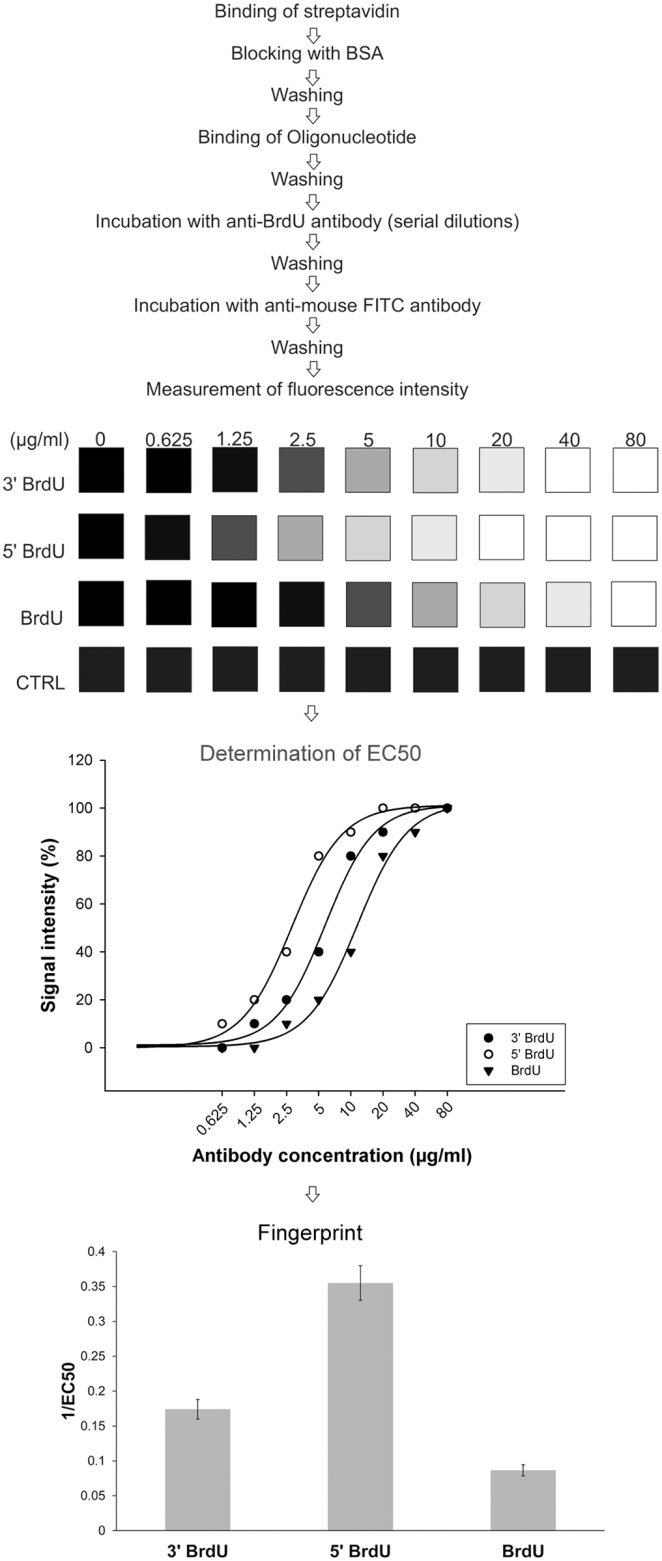
Scheme of the system for the antibody analysis. The scheme of the system for the antibody analysis is shown. The signal intensities are normalised to the signal obtained with the highest antibody concentration equal to 100%.

We measured the signal produced by BrdU-labelled oligonucleotides and subtracted the signal of the control oligonucleotide for every antibody concentration. Next, we constructed the curves showing the course of the dependence of the signal on the antibody concentration and determined the concentration when the signal was equal to the half of the saturated signal (EC50). The schema of the complete protocol including the method of signal evaluation is shown in Fig 1.

### The affinity to BrdU strongly depends on BrdU position for most of clones tested

**Fig 2:**
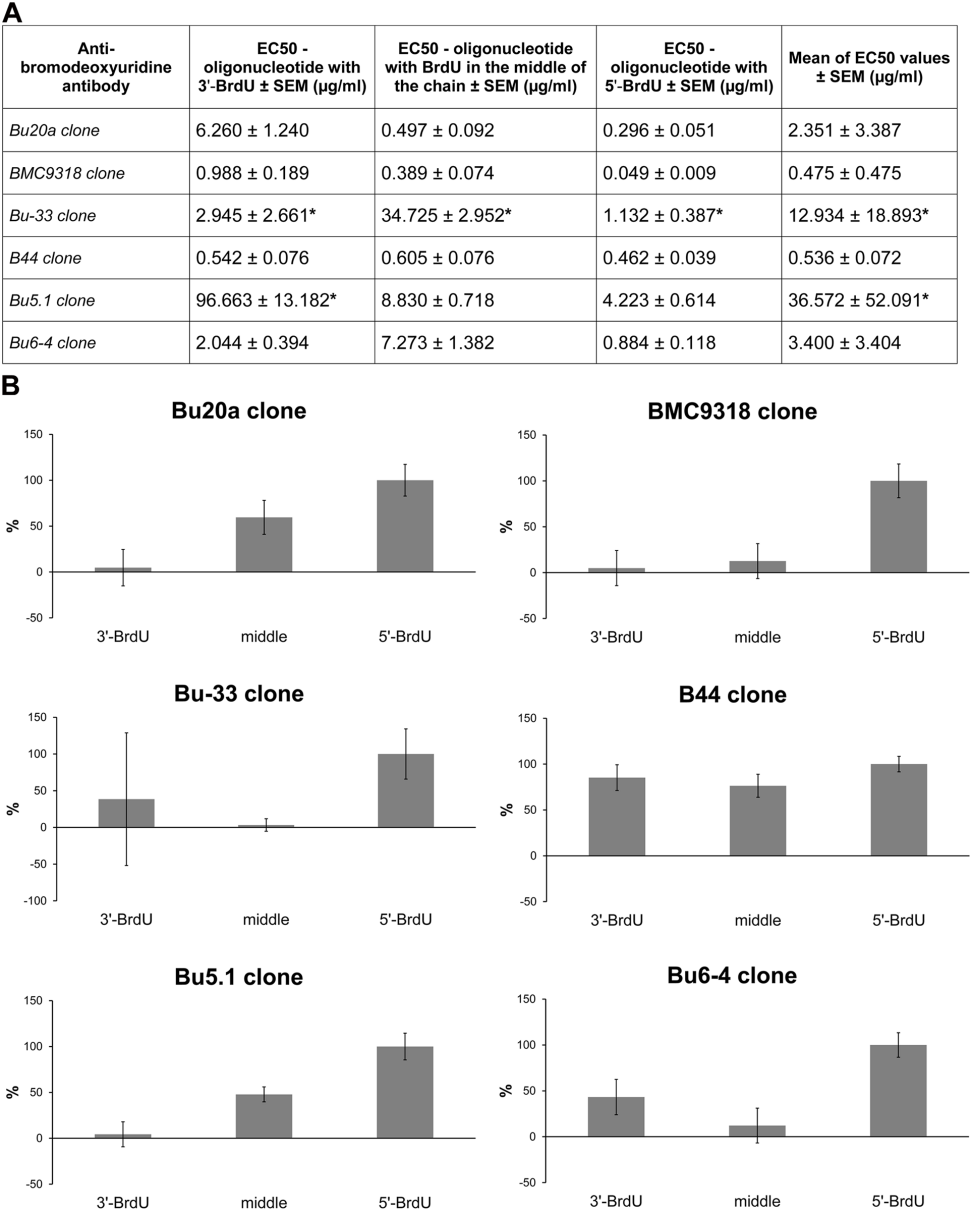
The EC50 values (A) and fingerprints (B) of the affinity of the anti-bromodeoxyuridine antibodies. (A) The EC50 values for three oligonucleotide probes containing BrdU at the 3' or 5' end or in the central part of the oligonucleotide chain ± SEM are shown for each antibody. In the last column, the mean of these three EC50 values ± SEM is shown. The values marked with a star were estimated and probably are below the real values. (B) The fingerprint involving the EC50 values for the oligonucleotides containing BrdU at the 3' or 5' end or in the central part of the oligonucleotide chain are shown in a bar graph. The affinity of the clones is expressed as 1/EC50 ± SEM in percent. The data are normalized to the signal obtained for the oligonucleotide with BrdU at the 5' end. This signal is equal to 100%.

The data showed significant differences in the affinity of antibody clones to BrdU. The values of EC50 differed both between the clones and between the oligonucleotides used (Fig 2A). We found that the clone Bu-33 showed such low affinity to BrdU that the antibody concentrations used did not enable us to determine the concentration resulting in the saturation of the signal for any oligonucleotide used. Concerning the clone Bu5.1, we did not reach the saturation concentration for the oligonucleotide containing BrdU at the 3' end. In these cases the EC50 value was calculated from the maximal measured signal (clone Bu-33) or from the average saturation signal measured for oligonucleotide with BrdU in the central part or at the 5' end of the chain (clone Bu5.1). The combination of the three measurements of EC50 for BrdU-labelled oligonucleotides provided a pattern (fingerprint) that reliably identified the given antibody clone (Fig 2B). We used 1/EC50 ratio in this case as it is directly proportional to the antibody affinity.

**Fig 3:**
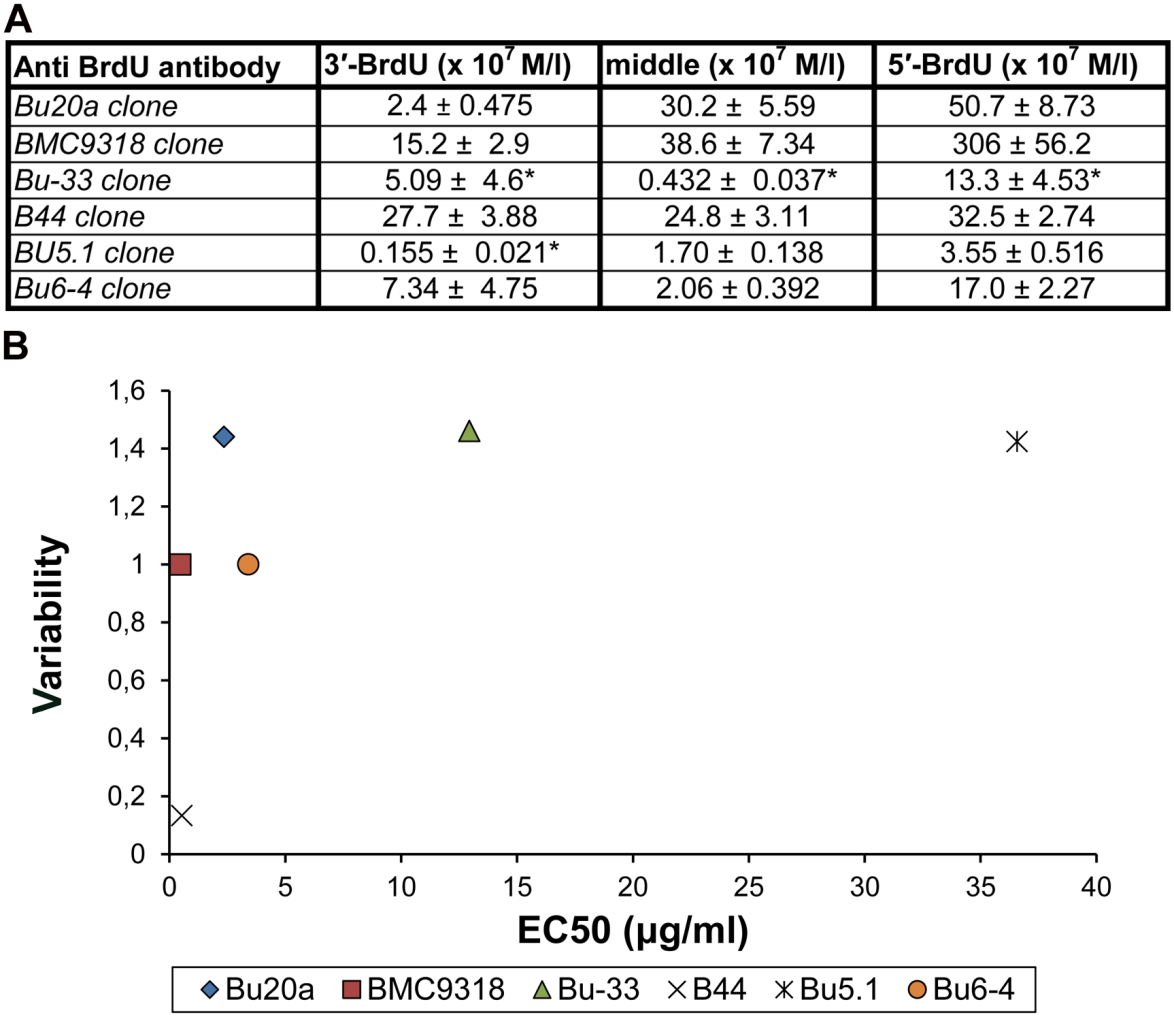
Affinity constants (A) and the scatter plot (B) of tested antibody clones. (A) The affinity constants for anti-bromodeoxyuridine antibody clones and oligonucleotides containing BrdU at the 3' or 5' end or in the central part of the oligonucleotide chain are shown. The values marked with a star were estimated and probably are above the real values. The data are presented as the mean ± SEM. (B) The scatter plot shows the distribution of the anti-bromodeoxyuridine clones with respect to the mean and variability of the EC50 values calculated from the EC50 values for oligonucleotides containing BrdU at the 3′ or 5′ end or in the central part of the oligonucleotide chain. The variability is expressed as the ratio between the standard deviation and the mean of the EC50 values and is plotted against the EC50 mean.

All of the tested clones exhibited the highest affinity to the oligonucleotide with BrdU at the 5' end (Fig 2B). Three clones exhibited the lowest affinity to the oligonucleotide with BrdU at the 3' end and three clones to the oligonucleotide with BrdU in the central part of the chain. The lowest EC50 value (0.049 μg/ml) and therefore the highest affinity to the oligonucleotide with BrdU at the 5' end was exhibited by the clone BMC9318 (Fig 2A). As the molecular weight of IgG is ~ 150,000 [25], it corresponds to the concentration about 3.23 × 10^−10^ M/l. Considering the fact, that EC50 is equal to the concentration when the oligonucleotide with BrdU is occupied from the one half by the antibody and the fact that the affinity constant K is the reciprocal of the concentration of the antibody at which the antigen is half saturated, the affinity constant is approximately 3.06 × 10^9^ M^-1^. The overview of the measured affinity constants for the particular antibody clones is in Fig 3A. As the high number of measurements does not enable a clear comparison between the clones, we plotted the average value of EC50 against the ratio of the standard deviation and the average value of EC50 (Fig 3B). From the graph in Fig 3B, it is obvious that the clone B44 exhibited the lowest variability and concurrently a high average affinity. The next two clones, BMC9318 and Bu6-4, exhibited similar variability, but a different average of EC50. The last three clones tested exhibited similar variability but a very different average of EC50. In the case of the clones Bu-33 and Bu5.1, we were not able to determine the exact EC50 values in all or in one oligonucleotide, respectively, and the stated EC50 values are the minimal EC50 values.

**Fig 4:**
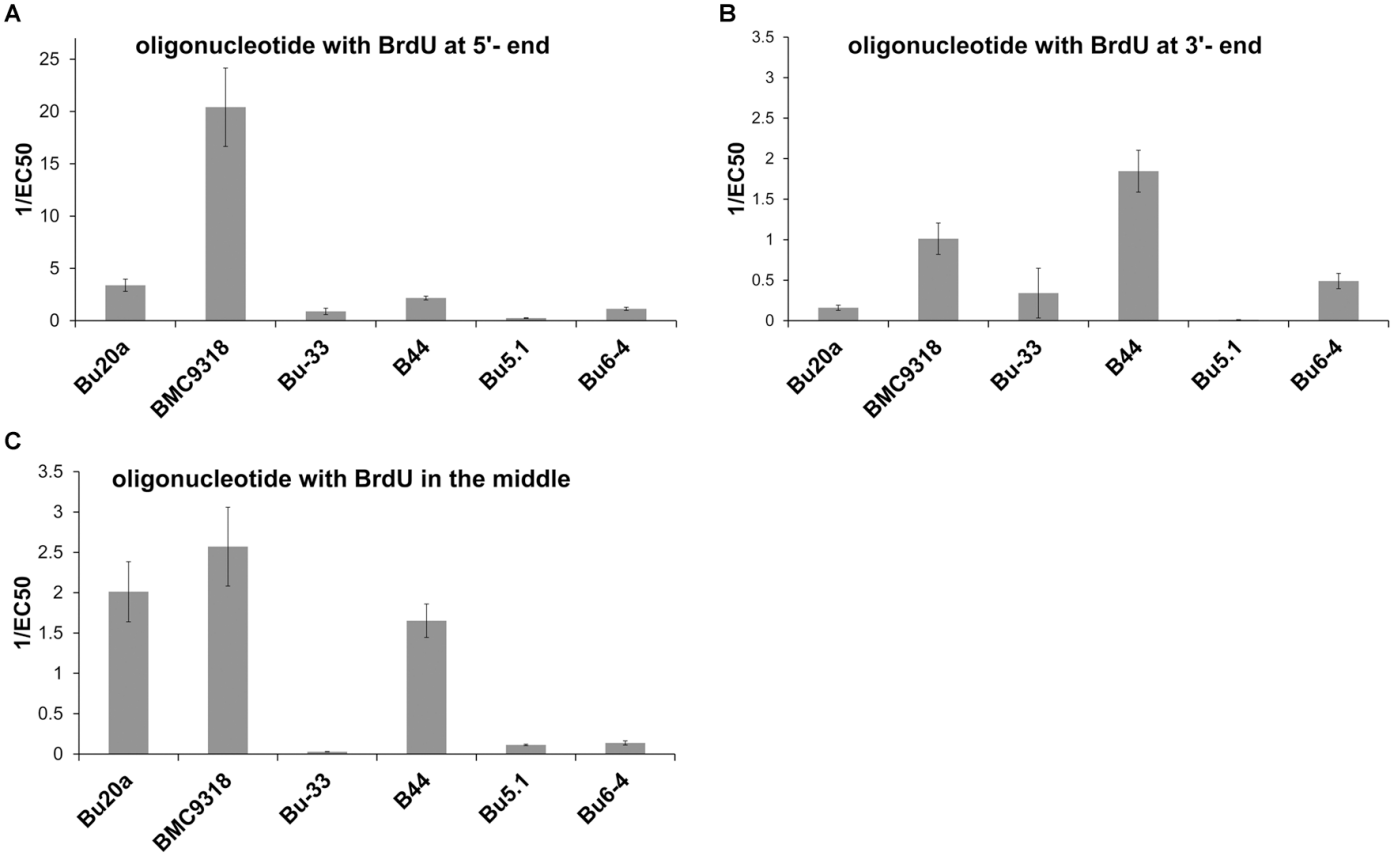
Comparison of the affinities of the anti-bromodeoxyuridine antibodies with respect to the BrdU position. The affinities of the antibody clones expressed as 1/EC50 ± SEM for the oligonucleotides containing BrdU at the 5' (A) or 3' (B) end or in the central part of the chain (C) are shown.

Our data can be also used for the estimation of the real orientation of BrdU after various treatments. For instance, if the orientation of BrdU in the cellular DNA after the BrdU revelation step resembles the orientation of BrdU at the 5' end of the oligonucleotide probe, the highest signal can be definitely expected if the clone BMC9318 is used (Fig 4A). If the orientation resembles rather the situation in the oligonucleotide with BrdU at the 3' end, the best signal should be provided by the clone B44 (Fig 4B). In the case of the orientation of BrdU similar to that in an oligonucleotide with BrdU in the central part of the chain, the clone BMC9318 should provide the highest signal (Fig 4C).

### The clones B44 and BMC9318 provide satisfactory results in most situations

**Fig 5:**
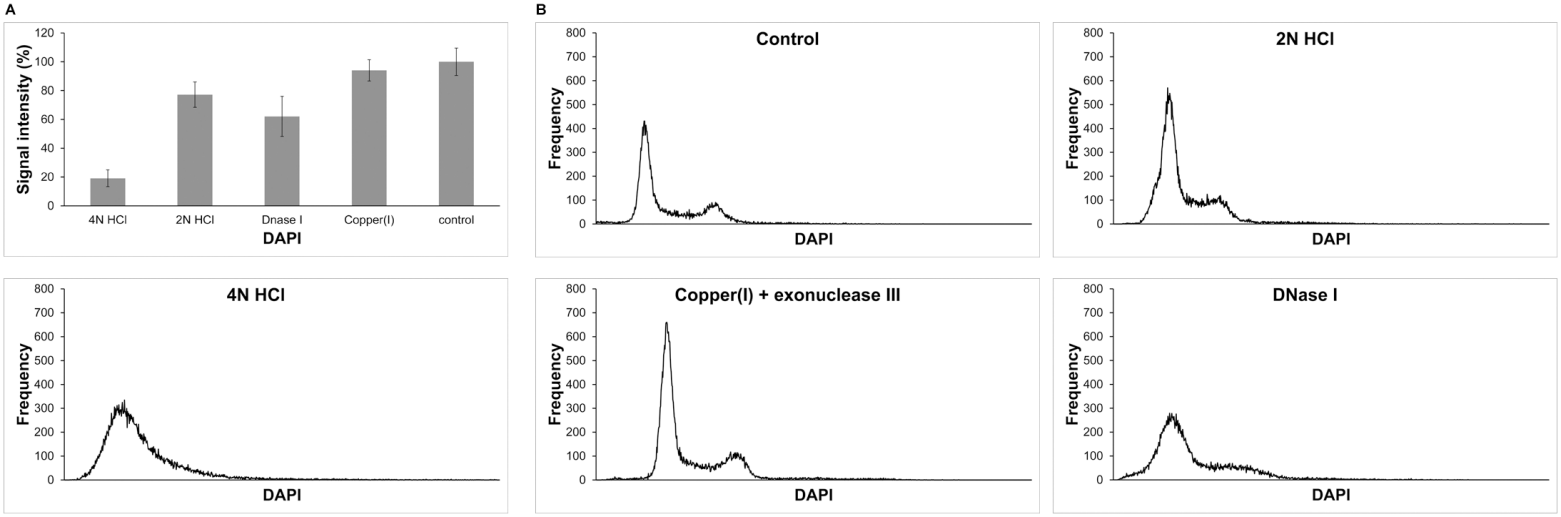
The effect of various treatments on the DNA content (A) and DNA histogram (B). (A) The DAPI signal in cell nuclei after the treatment with 2N HCl, 4N HCl, DNase I, copper(I) ions with exonuclease III and in the control cells is shown. DAPI signal of the control non- treated cells is equal to 100%. The data are normalized to % of the signal of control cells. The data are shown as the mean nuclear signal ± SEM. (B) The histograms of nuclear integral DAPI signal in non-treated cells (control) or cells treated with 2N HCl, 4N HCl, copper(I) ions and exonuclease III or DNase I is shown.

Next, using the image cytometry we analysed the signal after the detection of BrdU incorporated in cellular DNA. We compared four different protocols of BrdU detection. Two protocols used hydrochloric acid, one uses DNase I and one uses the oxidative degradation of DNA and the action of exonuclease III to reveal BrdU in DNA. The cells were incubated with 10 μM BrdU for 1 hour, fixed and permeabilized. The extent of DNA degradation was monitored by DAPI staining (Fig 5A). The lowest damage of DNA was observed in the protocol based on the monovalent copper ions, followed by the protocols based on 2N HCl, DNase I and 4N HCl.

In order to analyse the effect of different BrdU revealing protocols on the DNA profiles, we analysed ca 20,000 nuclei from each experiment and constructed the corresponding histograms (Fig 5B). We observed two peaks corresponding to the fraction of G1 and G2/M cells separated by the fraction corresponding to the S phase cells in the case of the control cells and cells treated with copper(I) ions and exonuclease III. The treatment with 2N HCl substantially lowered the peak corresponding to the G2/M cell fraction. The DNase I treatment resulted in the complete disappearance of the area corresponding to the G2/M cell fraction and extended the area corresponding to the G1 fraction. In the case of 4N HCl, we observed only one peak.

For the signal maximisation and background minimisation, we improved the protocol of the oxidative degradation of DNA compared to the originally published protocol [5]. All the protocols used are described in detail in the Material and Methods section. In all of the cases, we used the same concentration of anti-BrdU antibodies (5 μg/ml).

For the mutual comparison of signal intensity, we used the ratio between the signal with the background (SB) and the background (B). The SB/B ratio is directly proportional to the specific signal provided by antibody. This method enabled us to use the same sample for both measurements. The same settings of the microscope and camera including the acquisition time were used for the measurement of the signal intensity for the same BrdU revealing protocol. The acquisition time was adjusted according to the antibody that provided the highest signal for the protocol tested to the level that did not result in the saturation of the signal in any area of the sample.

More than 40% of the cells showed replicational activity after a 1-hour pulse with BrdU. In order to assure that only BrdU-positive cell nuclei are evaluated, we measured the mean nuclear signal intensity only in 30% of the highest labelled cells. This value included both the signal and background. For the background measurements, we used the average value calculated from the mean values of 40% of the lowest labelled cells. We analysed 5,000 cells for every sample and every experiment was performed in triplicate. The control samples were incubated in medium without BrdU.

**Fig 6:**
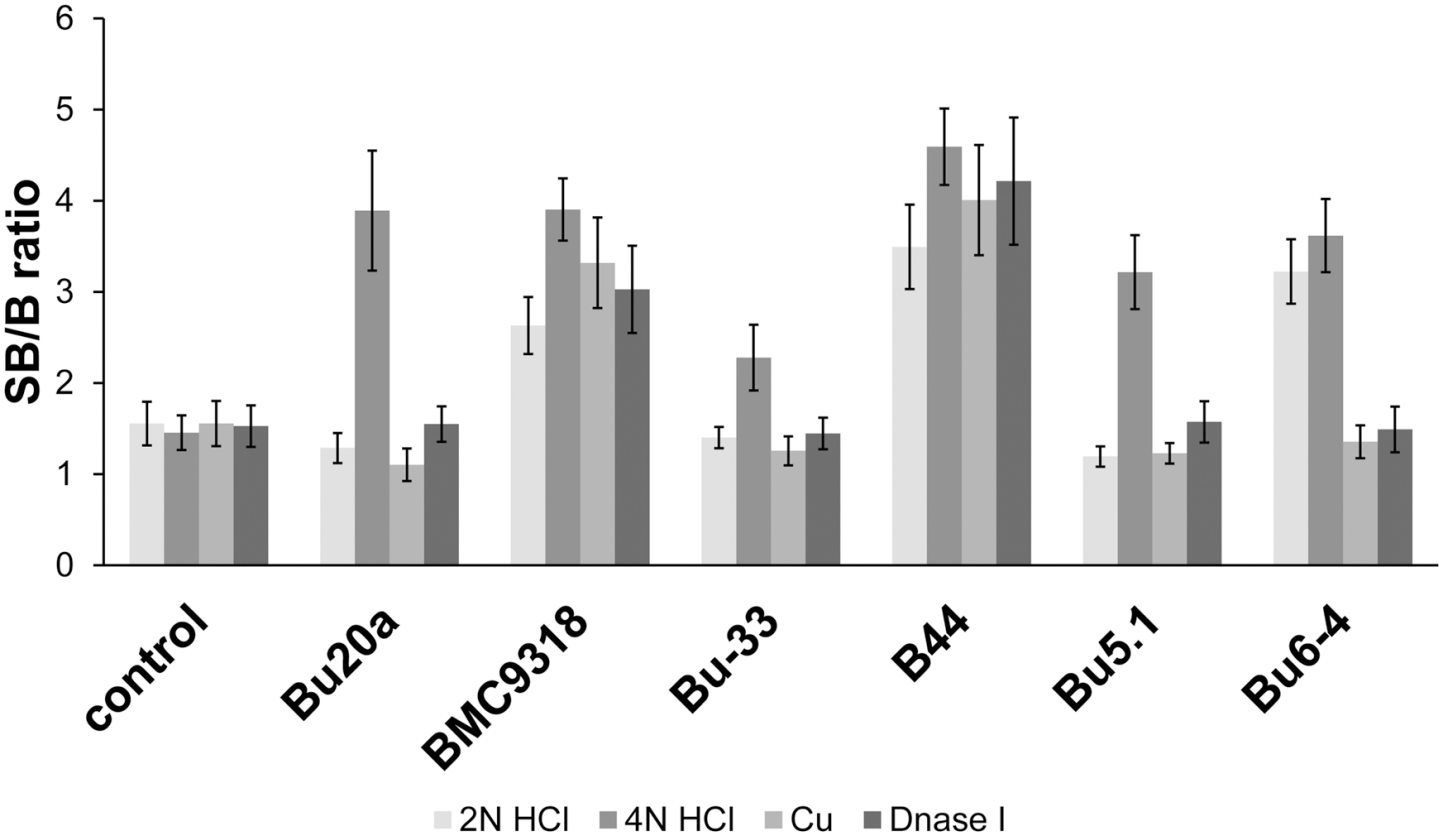
The effect of BrdU detection protocol on the affinity of the anti-bromodeoxyuridine clones to BrdU. The analysis of the signal in the cells after a 1-hour incubation with BrdU or in the control, BrdU non-labelled, cells is shown. BrdU was revealed with 2N HCl, 4N HCl, DNase I or copper(I) ions with exonuclease III. The signal intensity is plotted as the SB/B ratio ± SEM. (signal with the background/background).

We found that after the treatment with 4N HCl, all of the antibody clones reacted with BrdU (Fig 6). The highest SB/B ratio was provided by the clone B44, the lowest by the clone Bu-33. Only this protocol assured that the values of SB/B ratio were significantly higher than the ratio measured in the control cell for all the antibodies tested. In the rest of the protocols used, we observed a dramatic decrease of antibody clones that were able to react significantly with the incorporated BrdU. While in the case of 2N HCl, the B44, BMC9318 and Bu6-4 clones provided a significant BrdU signal, only the clones B44 and BMC9318 provided the significant signal in the case of DNase I and copper(I) treatment (Fig 6).

Interestingly, both clones (B44 and BMC9318) exhibiting the strongest affinity to oligonucleotide with BrdU at the 3' end (Fig 4B) were able to recognize incorporated BrdU in all the used protocols of BrdU detection. On the other hand, the clone Bu6-4 which provided the third strongest signal with oligonucleotide with BrdU at the 3' end (Fig 4B) provided a signal only after treatment with 4N and 2N HCl. In this respect, the graphic analysis of individual fingerprints (Figs 2B and 3B) seems to provide better guidance for the assessment of antibody affinity to BrdU under various conditions than the evaluation of the affinities to individual oligonucleotides.

### IdU provides a higher SB/B ratio than BrdU in the case of the clone B44

**Fig 7:**
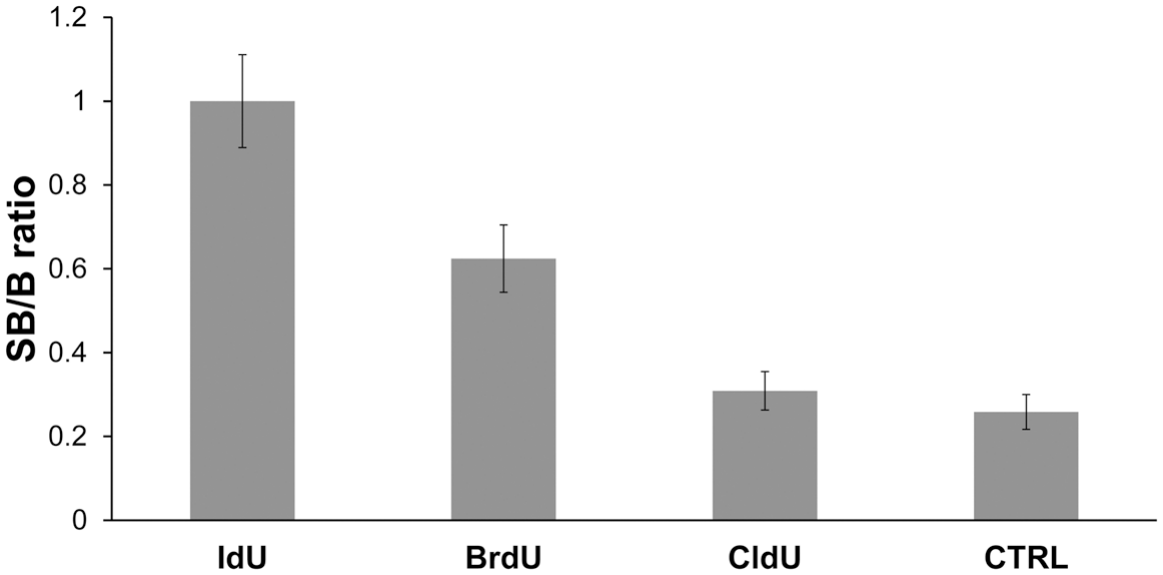
The comparison of BrdU, IdU and CldU affinities for the anti-bromodeoxyuridine antibody clone B44. The analysis of the signal in cells incubated for 1 hour with BrdU or IdU or CldU or in the control, non-labelled, cells. The copper(I) ions and exonuclease III was used for BrdU/CldU/IdU revelation. The values are normalised for the IdU signal equal to 1. The data are presented as SB/B ± SEM (signal with the background/background).

Although the antibody clone B44 was selected after the immunization of mice with iodouridine-conjugated ovalbumin, it is usually used for the detection of BrdU. As it is not completely clear if the affinity of this clone to IdU is significantly higher to IdU than to BrdU, we tested IdU instead of BrdU for the detection of DNA synthesis. We used the protocol based on the monovalent copper treatment. This protocol is mild with respect to DNA destruction and provides histograms of DNA content similar to the control cells. This is an important supposition of reliable cell cycle analysis. Also, the effect of the copper(I) treatment on the protein components is relatively low [5]. We incubated cells either with BrdU, IdU or CldU (CldU is modified nucleoside that can be used as a marker of DNA replication as well; [26,27]) and analysed the SB/B ratio (Fig 7). It is evident that the normalised SB/B ratio for IdU is significantly higher than that for BrdU (Fig 7). The signal provided by CldU was almost at the level of the control cells. The very low signal provided by CldU is in agreement with the fact that the clone B44 is used in the double labelling experiments where samples are labelled with IdU and CldU [27–30].

**Fig 8:**
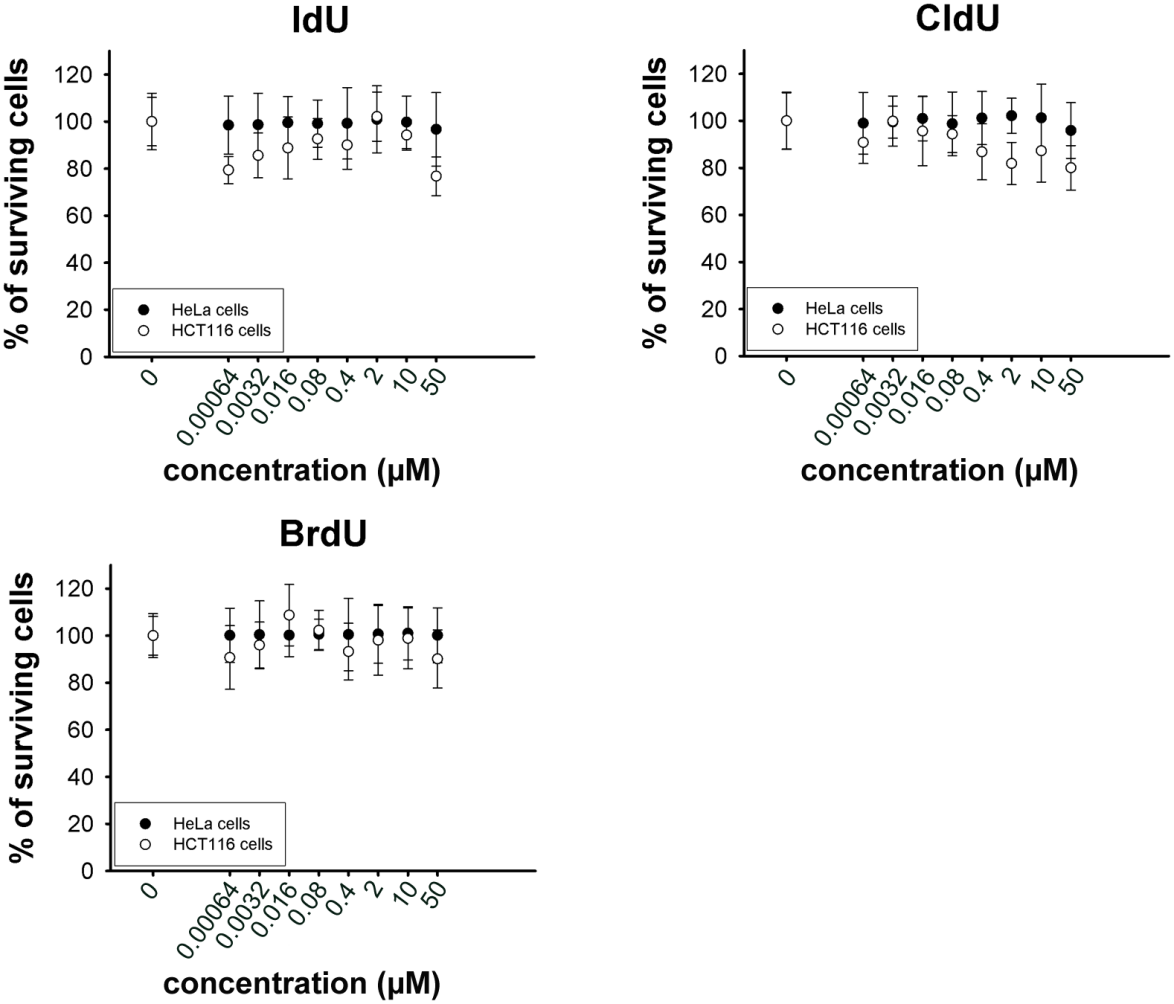
MTT assay results. The results of the MTT assay are shown. The impact of BrdU, IdU and CldU on HeLa and HCT116 cells is shown. The data represents the mean ± SEM.

The performed analyses of cytotoxicity (MTT assay) showed that BrdU, IdU and CldU exhibited low if any cytotoxicity for the concentrations that are usually used for the detection of replicational activity in both cell lines tested (HeLa and HCT116 cells, Fig 8).

**Fig 9:**
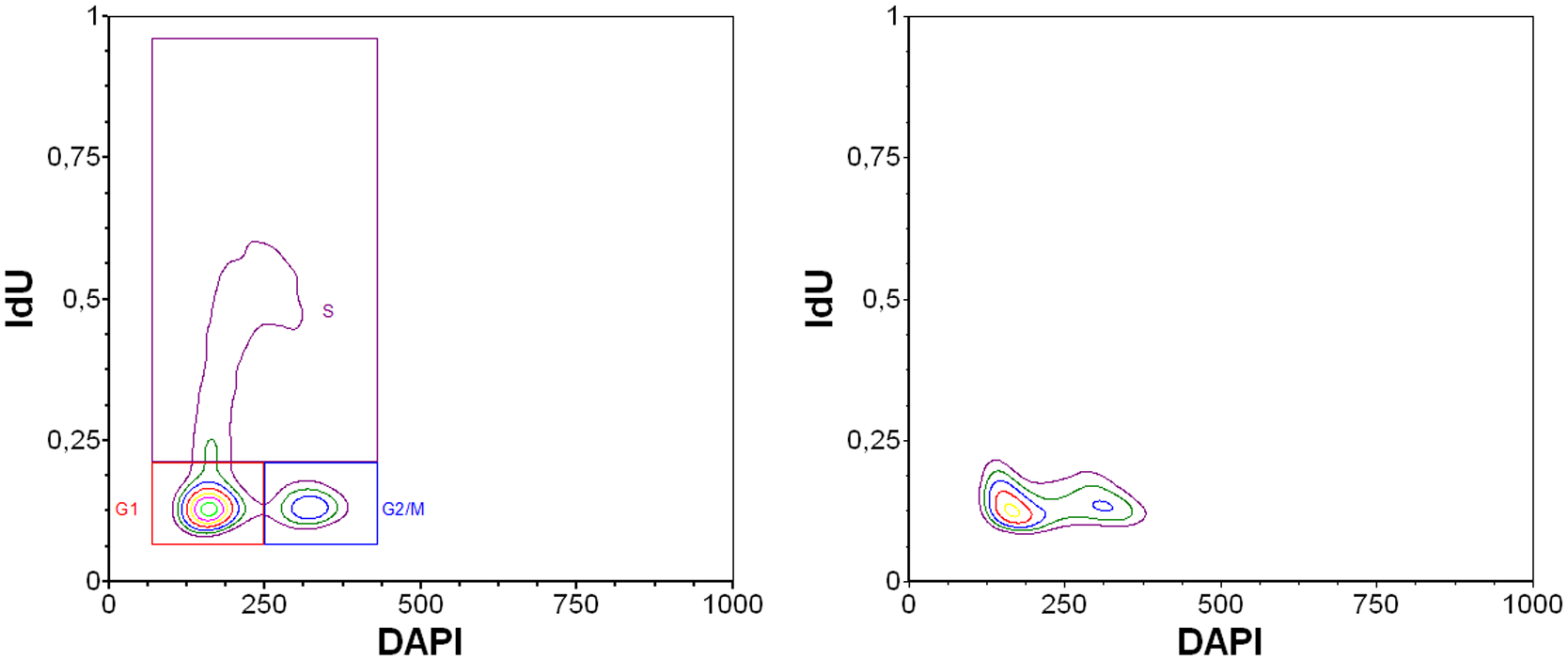
The bivariate analysis of the IdU signal in HeLa cells. The bivariate analysis of the replication signal in HeLa cells after a 20-minute incubation with 10 mM IdU (A) and in control, non-labelled HeLa cells (B) is shown. The copper(I) ions and exonuclease III was used for IdU revelation in DNA. The percent of cells in G1 phase was ~50%, in S phase ~36% and in G2/M phase ~14%.

As the short pulses are generally preferred in many situations including cell cycle analysis, we used a 20-minute labelling of HeLa cells by IdU instead of a 1-hour labelling and concurrently performed the analysis of the cell cycle using a bivariate analysis of DNA content and replicational signal (Fig 9). It is clear that the bivariate analysis allowed a good differentiation of the particular phases of the cell cycle using image cytometry.

## Discussion

In the study presented, we developed a system for the anti-bromodeoxyuridine antibody analysis and performed analysis of six anti-bromodeoxyuridine antibody clones. The results of the analysis were compared with the results of four protocols for the detection of BrdU *in situ*.

The system developed is based on the analysis of the affinity of the antibody clones to the anchored oligonucleotides containing BrdU at three different positions. BrdU was alternatively placed at 5' or 3' or in the central part of the oligonucleotide chain. We determined the EC50 using two fold serial dilutions of antibodies and fluorescent signal provided by secondary antibody conjugated with Alexa Fluor 488.

Clones differed one from another by the values of EC50 substantially. These differences were clearly seen in scatter plot where the position of antibody is defined by the value of the ratio between the standard deviation and the mean of EC50 of oligonucleotides used and by the EC50 mean of these oligonucleotides.

The low average EC50 is evidently a very important prerequisite for a strong reaction with BrdU in situations when BrdU orientation is a mixture of orientations resembling those tested. On the other hand, the variability between the measurements for BrdU in different positions can play an important role in situations when one BrdU position prevails or the orientation is different from those tested. Apparently, the lower variability means a lower dependence on orientation of BrdU. Generally, the clones with the high affinity (low EC50 mean) and low variability should provide the highest signal *in situ* and this signal should be less dependent on the differences between protocols of BrdU detection in DNA than the clones with the high signal variability.

In this respect, we tested four different protocols for BrdU detection—protocol using 2N and 4N HCl, copper(I) treatment and DNase I treatment. The protocols used differed in the mechanism and the type of DNA damage they produce. The use of hydrochloric acid leads to the depurination and gradual destruction of DNA chains [7]. DNase I produces the high number of single- and double-stranded breaks and probably single-stranded areas as well [31–33]. Oxidative degradation of DNA using monovalent copper leads to the creation of breaks followed by the creation of single-stranded areas that probably represent the prevalent damage of DNA [5,34].

According to our results, protocol based on 4N HCl resulted in the highest damage of DNA and completely disabled analysis of DNA content. This protocol is also inconvenient for the detection of RNA or protein components [5]. However, this protocol represents the only protocol providing a significant signal with all of the anti-bromodeoxyuridine antibody clones. In the rest of protocols used, the ability to recognize incorporated BrdU was decreased.

Only two clones were able to react with BrdU in all four protocols: clones B44 and BMC9318. This finding was in accordance with our suggestion that the clones with the highest average affinity (the lowest EC50 mean) and concurrently with the lowest variability can provide the highest signal *in situ*. It indicated that the antibody clones with the high affinity to cellular DNA under various situations should be close to the clones B44 and BMC9318 in the graph area (Fig 3B).

Furthermore, it seems that the BrdU orientation in cellular DNA, especially after the mild destruction of cellular DNA, is different from those in the oligonucleotides tested. For example, after a copper(I) and exonuclease treatment that reveals BrdU in the central part of DNA a positive and strong reaction with the clone Bu20a can be anticipated. However, this clone did not provide any significant signal under these conditions. It is not a surprising finding as the chromatin structure is very complicated when compared to synthesised oligonucleotides.

Although the clone BMC9318 provided signal in all tested situation and in this respect, provided similar results as the clone B44, the clone B44 provided higher specific signal in all protocols tested. In addition, our results clearly showed that the specific signal can be further increased if IdU is used instead of BrdU.

According to the MTT assay performed, even 50 μM concentrations of BrdU, IdU or CldU did not result in a substantial decrease of cell proliferation with respect to the non-treated cells. In this respect, we previously reported that the IC50 value for EdU was 0.429 ± 0.016 μM for HeLa cells and 6.6 ± 2.328 μM for HCT116 cells [11]. In the case of BrdU, IdU and CldU, the IC50 concentration are clearly much higher than for EdU. It is evident that although the halogenated deoxyuridine analogues can be replaced by EdU in many situations, their use is clearly advantageous in the case of long-term experiments as their toxicity is much lower as compared to EdU.

Taking all the results together, we can conclude that:

1. The developed system enables identification of the most efficient clones for the detection of anti-bromodeoxyuridine antibody *in situ*.
2. Only the clones B44 and BMC9318 can be used in all BrdU detection protocols tested
3. The clone B44 is superior to the clone BMC9318 as it provided higher specific signal in all protocol tested.
4. The clone B44 exhibited higher *in situ* specific signal if IdU is used instead of BrdU.
5. The BrdU detection protocol based on 4N HCl provides the highest specific signal for all antibodies and is convenient in situations when there is no requirement for the cell cycle analysis and/or simultaneous localization of RNA or protein components.
6. The protocol based on copper(I) ions provides the most powerful protocol for the image cytometry evaluation of the cell cycle. It exhibits very mild effect on DNA content. As this protocol enables also simultaneous localization of protein and RNA components [5], it can be generally used for multi-parametric studies.
7. The protocol based on the DNase I treatment is not suitable for the cytometric analysis because of its relatively high DNA destruction. In this respect, the protocol based on 2N HCl treatment provided better results. On the other hand, DNase I treatment does not result in destruction of RNA and protein components and allows co-localization of DNA replication activity and RNA or protein components (e.g. [35]).
